# Books and Magazines

**Published:** 1901-08

**Authors:** 


### Books and Magazines.

SAUNDERS' QUESTION COMPENDS.—Essentials of refraction and of diseases of the eye. By Edward Jackson, A. M., M. D., Emeritus Professor of Diseases of the Eye in the Philadelphia Polyclinic. Third edition; revised and enlarged; 12mo.; 261 pages; 82 illustrations. Philadelphia and London: W. B. Saunders & Co. 1901. Cloth, $1.00, net.

This compend will be found to meet the needs of the general practitioner and the beginner in ophthalmic work quite as well, if, indeed, not better, than almost any of the larger and more expensive works on the subject. The author has added in the third edition a description of the tests of vision required in the army, navy, railway services, and public schools, which serves to make quite plain the mode of their application.

The Care of the Baby.—A manual for mothers and nurses, containing practical directions for the management of infancy and children in health and in disease. By J. P. Crozer Griffith, M. D., Clinical Professor of Diseases of Children in the Hospital of the University of Pennsylvania, Philadelphia. Second revised edition; 12mo.; 404 pages, with 67 illustrations in the text and five plates. W. B. Saunders & Co., 925 Walnut street, Philadelphia. 1898. Price, $1.50, net.

Dr. Griffith has written one of the best books on this subject that has yet appeared. In his second edition he has enlarged the work considerably, and with his usual painstaking and careful precision has submitted it to a complete revision. The book is a very practical one, and covers thoroughly what it undertakes to do on its title page. The author lays down clearly the lines that the parent or nurse must follow in the care of the child, with safety and without usurpation of the place of the physician. A dissemination of the knowledge contained in the book would often lead to medical consultation in instances in which it is now neglected through ignorance or indifference. The book is altogether a useful and most commendable one, not alone for mothers and nurses, but for medical students and practitioners as well.

SENN'S PRACTICAL SURGERY.—Practical surgery. A work for the general practitioner. By Nicholas Senn, M. D., Ph. D., LL. D., Professor of Surgery, Rush Medical College, Chicago. Handsome octavo volume of 1133 pages, with 650 illustrations, many in colors. Philadelphia and London: W. B. Saunders & Co. 1901. Cloth, $6.00, net.

This work represents the surgical experience of its distinguished author during a period of twenty-five years of active practice. It is as its titles implies, essentially a practical work on surgery, and it has been prepared with special reference to the needs of the general practitioner. All of the emergency operations which the physician in general practice is likely to be called upon to do are carefully described and fully illustrated. The subjects of most importance are given due prominence, some of them being treated at great length. The sections on shock, hemorrhage, wounds, fractures, dislocations, and amputations, and those on intestinal surgery, have received the greatest amount of attention. The work does not cover the entire field of surgery, which fact adds to its value, since its limited scope allows the author to devote the attention to the principal chapters which, in his opinion, is warranted by their importance.

Refraction, and How to Refract.—Including sections on optics, retinoscopy, the fitting of spectacles and eye-glasses. By James Thorington, A. M., M. D., Professor of Diseases of the Eye in the Philadelphia Polyclinic and College for Graduates in Medicine; Associate Member of the American Ophthalmological Society; Fellow of the College of Physicians of Philadelphia, etc. Second edition; 200 illustrations, 13 of which are colored. Philadelphia: P. Blakiston's Son & Co., 1012 Walnut street. 1900. Price, $1.50.

This is a book full of practical ideas, systematically arranged, and its author seems to have spared no pains, and has exercised much skill in his selections of illustrations.

The book is not large, yet it will be found to be sufficiently comprehensive to serve every purpose of the optician.

A second edition having been called for within so short a time (the first edition was published in November, 1899) speaks for the popularity of the book; and in this the second edition the author has given us a thorough revision of the book. Many of the illustrations are original ones, and are specially adapted for the purpose which they are supposed to serve.

In short, the book is all that is claimed for it, and we predict for it a continued ready sale.

DISEASES OF THE EYE.—A hand-book of ophthalmic practice. By G. E. de Schweinitz, M. D., Professor of Ophthalmology in the Jefferson Medical College, Philadelphia. Royal octavo; 696 pages; 256 illustrations, and three chromo-lithographic plates. W. B. Saunders & Co., 925 Walnut street, Philadelphia. 1899. Price, cloth, $4.00, net; sheep or half Morocco, $5.00, net.

The present edition of this well-known text book is brought up to date by the addition of a not inconsiderable amount of new matter and a thorough revision of the old. It is a beautifully printed and richly illustrated work, and is certainly well adapted for the use of students since it is very plainly and practically written by one who has had large experience in teaching, and is familiar with the conditions which he describes.

Chapter I, on general ophthalmological principles, is worthy of special commendation. The theoretical part of the subject is so carefully explained and illustrated so finely that it makes it quite easy to grasp this difficult subject. The same can be said in reference to the second chapter, and especially in regard to functional testing. The description of the use of the perimeter is one of the best that has been written, and will be appreciated by every student who finds so much difficulty in the use of this instrument.

Although many text-books have been written upon the subject of skiascopy alone, it would be difficult, indeed, to find one condensing the whole subject in such small space and in so practical a manner as Dr. de Schweinitz does in Chapter III.

One of the best written chapters in the book is that upon glaucoma. The illustrations are fine, and the descriptions of the various forms of the disease thorough.

Chapter XXII, on operative ophthalmology, with the illustrations demonstrating the technique of the various operations, is undoubtedly the best that has been seen in any text-book.

The whole book is magnificent, wonderfully exempt from typographical errors, and the medical students of the United States, with this work in their hands, and the larger text-book on the eye, ear, nose and throat by Drs. de Schweinitz and Randall, have their ophthalmic education very completely provided for.

---

An American Text-book of Genito-Urinary Diseases, Syphilis and Diseases of the Skin.—By forty-seven specialists. Edited by L. Bolton Bangs, M. D., Professor of Genito-Urinary Surgery, University and Bellevue Hospital Medical College, New York, and W. A. Hardaway, A. M., M. D., Professor of Diseases of the Skin and Syphilis in the Medical Department of Washington University, St. Louis. Imperial octavo of 1229 pages, with 300 engravings and 20 full-page colored plates. W. B. Saunders & Co., 925 Walnut street, Philadelphia. 1898. Price, cloth, $7.00, net; sheep or half Morocco, $8.00, net.

This magnificent book is the joint work of a number of contributors, recognized authorities upon the subjects of which they treat, who have been allowed free expression of their individual opinion by the editors. The names of the authors cannot fail to give the volume a place of importance in surgical literature quite apart from its intrinsic value, and notwithstanding the articles vary widely in their standard of excellence, the general result is good, and is evidence that the editors have done their part conscientiously and well.

Of the first portion of the work, the largest and most important contribution is an article on the diseases of the male urethra, by Dr. G. Frank Lydson. This occupies nearly 130 pages, and the very full account of stricture deserves especial mention.

Dr. J. William White and Dr. Alfred C. Wood have contributed the article on diseases of the prostate, and describe very completely the advantages and disadvantages of castration for hypertrophy of the prostate, a method of treatment which was introduced by one of the authors (Dr. White).

The articles on syphilis occupy 140 pages, and have been written by seven authors. The description of the syphilitic diseases of the eye, and the general treatment of syphilis, deserve special commendation.

Every known skin disease is described clearly and precisely, and copiously illustrated. The space alloted to each skin affection is proportionated to its importance, and is never too long, and the text bears evidence that the editors have been judicious in their selection of authors.

The volume claims to be a complete and authoritative exposition of the diseases of the skin, and its execution justifies the claim. In every respect it is the very best work on genito-urinary diseases.

